# CNS methylation classifiers may misclassify normal developing cerebellar cortex as medulloblastoma

**DOI:** 10.1007/s00401-025-02907-1

**Published:** 2025-06-23

**Authors:** Jennifer A. Cotter, Alexander L. Markowitz, Everardo Castañeda, Chern-Yu Yen, Dejerianne Ostrow, Debra Hawes, Jianling Ji

**Affiliations:** 1 Department of Pathology and Laboratory Medicine, Children’s Hospital Los Angeles, Los Angeles, CA USA; 2 Department of Pathology, Keck School of Medicine, University of Southern California, Los Angeles, CA USA

Methylation profiling for tumor classification has entered mainstream neuropathology practice in recent years and now represents a key diagnostic modality in the field. However, DNA methylation changes are also instrumental in normal developmental biology, and complete representation of human developmental neuroanatomy is generally difficult to achieve in a reference set of normal control tissue. We recently discovered a concerning pitfall in the application of several robust central nervous system (CNS) tumor classifiers to developmentally normal, but immature, human cerebellar tissue.

The fetal and neonatal cerebellum contains an external granule cell layer (EGL) which constitutes a developmental niche for granule neurons [2, 6]. The EGL is transient during human neurodevelopment, but when present is highly proliferative (Fig. 1a–c). If not recognized as developmentally normal in a neonatal patient, the EGL can be mistaken as neoplastic on histologic examination, i.e., subpial growth of a CNS embryonal tumor such as medulloblastoma.

**Fig. 1:**
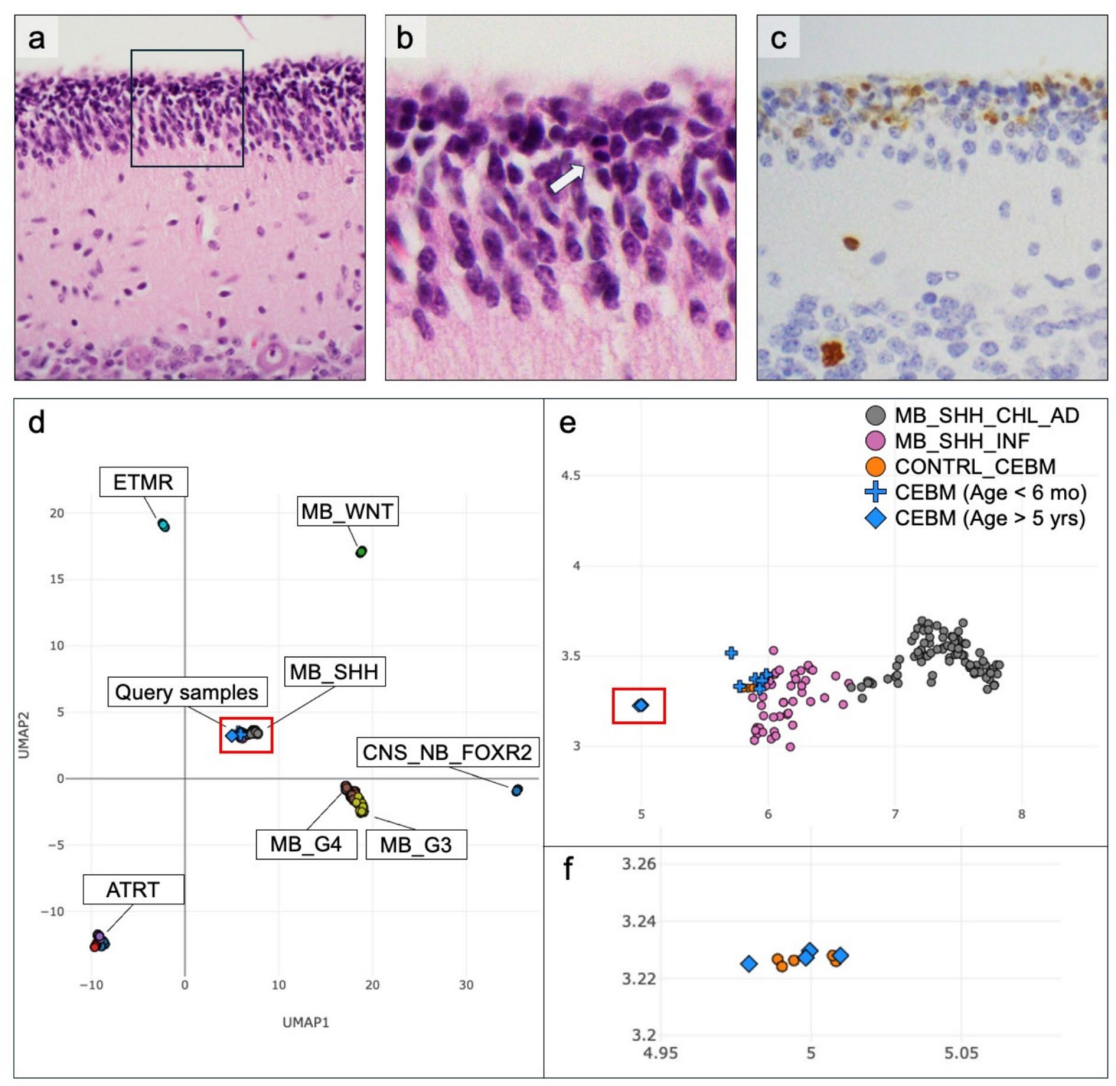
Appearance of cerebellar external granule cell layer and performance of methylation classification on cerebellar cortex samples before 6 months of age. In late gestation and infancy, the external granular layer (EGL) covers the cerebellar cortex, as seen in CHLA_CB_2 (**a**). The EGL consists of closely packed primitive-appearing cells with nuclear angulation (**b**, enlarged from boxed area shown in a) and, at times, irregular contours. Apoptotic bodies and mitotic figures (**b**, arrow) are common in the EGL. Ki-67 labeling index is elevated in the EGL due to ongoing cell proliferation (**c**). These features are all developmentally appropriate but can be misinterpreted as subpial spread of an embryonal neoplasm. Using DNA methylation array data, UMAP visualization (**d**–**f**) shows immature cerebellar tissue samples (blue crosses) more closely associate with MB_SHH groups (e, enlarged from boxed area shown in (**d**). Older pediatric samples (blue diamonds) show clustering away from those representing individuals aged < 6 months, and associate with reference controls (f, enlarged from boxed area shown in e)

DNA methylation array was performed using Infinium MethylationEPIC v1.0 and/or v2.0 on postmortem cerebellar cortex samples ranging from premature (28 gestational weeks) to postnatal 10 days and 4 months of age. Additionally, the study included three pediatric cases of 5 years, 16 years, and 17 years old. Postmortem samples were obtained from unrestricted autopsy cases with complete systemic and neuropathology examination; no malignancy was noted in any case. Macroscopically unremarkable cerebellum was sampled as per our hospital’s standard neuropathology protocol; specifically, samples are taken from the superior half of the left or right hemisphere, from cortex regions anterior to the primary fissure. Formalin-fixed, paraffin-embedded samples of cerebellar cortex were then processed according to manufacturers’ instructions (Zymo Research, Irvine, CA, and Illumina, San Diego, CA) and prepared as previously described [4]. All cases passed quality control metrics; each case showed a copy number profile without significant alterations. The resulting data were analyzed using our in-house classifier, Epignostix/DKFZ v12.8 and the NCI’s Bethesda v2.0 classifiers.

For infant samples through 4 months of age, classifiers either failed to match or indicated neuroblastic/embryonal tumors at the super-family/family level (Table 1). In all three classifiers, for all infant cases, a sub-threshold class score indicated a tumor entity, usually SHH-activated medulloblastoma (5/6 samples by NCI-Bethesda, 6/6 by Epignostix, and 4/6 by CHLA classifier). This is consistent with the observed overlap with medulloblastoma clusters by UMAP embedding (Fig. 1d–f). Two infant cases, CHLA_CB_2 and CHLA_CB_3, were called as match to medulloblastoma, SHH-activated by Epignostix with a high calibrated score (> 0.9). Samples from older children mostly aligned appropriately with established reference samples of control cerebellum, with the exception of the 5-year-old sample when run on EPIC v1.0, which also returned a sub-threshold result. EPIC v2.0 array of the same sample classified appropriately. UMAP embedding demonstrates a difference in clustering of methylation profiles between neonatal/infant patient samples and those from older children and teenagers (Fig. 1e–f). Of note, the original samples of cerebellar cortex used to develop the DKFZ classifier were obtained from adult tissue sources [4].

**Table 1:** Classifier results for pediatric cerebellar (non-tumor) samples of varying patient age. Bolded text highlights above-threshold results indicative of tumor, i.e., incorrect classification. Thresholds are classifier-specific. All infant samples failed to be correctly assigned to control tissue categories, across the three classifiers tested

Study ID (age)	array type	NCI Bethesda v2 family and class [score]	Epignostix CNS superfamily, family and class [score]	CHLA Classifier superfamily, family, and class [score]
CHLA_CB_1 (28 gestational weeks)	EPIC	**neuroblastic_embryonal_tumors [0.955]** no class match [MB_SHH_2, 0.18]	no superfamily match [MB, 0.7006] no family match [MB_SHH, 0.69569] no class match [MB_SHH_2, 0.66843]	no superfamily match [Glioma_glioneuronal, 0.65] no family match [Ependymoma, 0.5] no class match [Subependymoma, 0.97]
	EPICv2	**neuroblastic_embryonal_tumors [0.957]** no class match [MB_SHH_2, 0.178]	no superfamily match [MB, 0.53661] no family match [MB_SHH, 0.49513] no class match [MB_SHH_2, 0.66843]	no superfamily match [Glioma_glioneuronal, 0.48] no family match [Glioneuronal_neuronal, 0.58] no class match [Ganglioglioma, 0.93]
CHLA_CB_2 (term, 10 days)	EPIC	**neuroblastic_embryonal_tumors [0.966]** no class match [MB_G34_VII_0.108]	**MB [0.95636]** **MB, SHH [0.9473]** **MB_SHH_2 [0.91807]**	no superfamily match [Embyronal, 0.61] no family match [MB, 0.99] no class match [MB_SHH_INF, 0.94]
	EPICv2	**neuroblastic_embryonal_tumors [0.988]** no class match [MB_SHH_2, 0.384]	no superfamily match [MB, 0.76055] no family match [MB_SHH, 0.7093] no class match [MB_SHH_2, 0.58441]	no superfamily match [Embryonal, 0.61] no family match [MB, 0.99] no class match [MB_SHH_INF, 0.94]
CHLA_CB_3 (4 months)	EPIC	**neuroblastic_embryonal_tumors [0.938]** no class match [MB_SHH_2, 0.132]	**MB [0.92997]** **MB, SHH [0.91839]** no class match [MB_SHH_2, 0.88869]	no superfamily match [Embryonal, 0.89] no family match [MB, 0.99] no class match [MB_SHH_INF, 0.94]
	EPICv2	**neuroblastic_embryonal_tumors [0.92]** no class match [MB_SHH_2, 0.441]	no superfamily match [MB, 0.88273] no family match [MB_SHH, 0.85827] no class match [MB_SHH_2, 0.7944]	no superfamily match [Embryonal, 0.61] no family match [MB, 0.99] no class match [MB_SHH_INF, 0.94]
CHLA_CB_4 (5 years)	EPIC	**neuroblastic_embryonal_tumors suggestive [0.821]** no class match [ MB_G34_VII, 0.135]	no superfamily match [MB, 0.35719] no family match [MB_SHH, 0.20815] no class match [MB_SHH_2, 0.13745]	no superfamily match [Glioma_glioneuronal, 0.58] no family match [Glioneuronal_neuronal, 0.58] no class match [Ganglioglioma, 0.8]
	EPICv2	control_tissues [0.9] CONTR_CEBM [0.941]	no superfamily match [Control tissues, 0.70352] no family match [Control brain tissues, 0.70348] no class match [Control tissue, cerebellum, 0.70326]	no superfamily match [Control, 0.72] no family match [Control brain tissue, 0.93] no class match [Control tissue, cerebellum, 0.66]
CHLA_CB_5 (16 years)	EPICv2	control_tissues [0.98] CONTR_CEBM [0.99]	Control tissues [0.99777] Control brain tissues [0.99777] Control tissue, cerebellum [0.99777]	Control [0.91] Control brain tissue [0.95] Control tissue, cerebellum [0.83]
CHLA_CB_6 (17 years)	EPICv2	control_tissues [0.955] CONTR_CEBM [0.982]	Control tissues [0.96989] Control brain tissues [0.96989] Control tissue, cerebellum [0.96989]	no superfamily match [Control, 0.76] no family match [Control brain tissue, 0.93] no class match [Control tissue, cerebellum, 0.71]

SHH-activated tumors represent the most common medulloblastoma group among infant patients; however, the infant samples submitted in this study represent non-neoplastic, developmentally normal cerebellar cortex. The basis for the association of normal immature cerebellum with SHH-activated medulloblastoma is not entirely clear, but may be influenced by the differential expression of SHH pathway genes during normal cerebellar development, when SHH signaling from the Purkinje cells functions as a key driver of proliferation and differentiation for granule neurons [5, 9]. Of note, the proposed cell of origin for SHH-activated medulloblastoma is in the cerebellar granule neuron progenitor lineage[7, 8].

Our results demonstrate overlap in methylation profiles between normal developing cerebellum and medulloblastoma using current classifiers. In combination with a misinterpretation of the morphology and/or immunoprofile of the normal external granule cell layer, a spurious classifier result could prompt a misdiagnosis of medulloblastoma in an infant patient.

We acknowledge that current CNS tumor methylation classifiers are not designed or intended to perform on samples without tumor content. While it is unusual for a non-tumor sample to be submitted for methylation profiling, this can occasionally occur in a diagnostic setting, whether due to low tumor fraction, an error in sample preparation resulting in usage of an incorrect block, or potential pathologist misinterpretation of normal immature cerebellar histology. Accuracy of molecular assays in distinguishing tumor from non-tumor samples is increasingly relevant as intraoperative technologies emerge [3]. Furthermore, systematic molecular analyses of non-tumor samples from diverse anatomic regions and developmental ages may be needed, along with an algorithm training process targeting both cancer and control tissues, before optimal assay sensitivity and specificity can be achieved.

In summary, extra caution should be taken when evaluating DNA methylation classifier results for cerebellar samples from very young or premature infants, particularly if calibrated scores are below established thresholds. The misclassification we observed is recurrent across different classifiers using existing reference sets; however, opportunities to improve classifier performance exist. This error could be mitigated by integrating more diverse samples into the reference dataset, including those derived from normal control tissues, with associated data inclusive of patient age. This calls for improved warehousing of reference data to enable ongoing improvements for methylation classifiers [1]. In the meantime, we highlight these cases which we believe warrant caution in the interpretation or weighting of the results of methylation classification, which should always be performed in the context of routine expert histologic examination.

## Data Availability

DNA methylation array data derived from the cerebellar samples in this study are available via GEO (GSE299377).
